# Prognostic Nomograms Combined Adjuvant Lenvatinib for Hepatitis B Virus–related Hepatocellular Carcinoma With Microvascular Invasion After Radical Resection

**DOI:** 10.3389/fonc.2022.919824

**Published:** 2022-07-11

**Authors:** Shilei Bai, Lei Hu, Jianwei Liu, Minmin Sun, Yanfu Sun, Feng Xue

**Affiliations:** ^1^ Department of Hepatic Surgery II, the Eastern Hepatobiliary Surgery Hospital, Second Military Medical University (Naval Medical University), Shanghai, China; ^2^ Department of Hepatic Surgery I, the Eastern Hepatobiliary Surgery Hospital, Second Military Medical University (Naval Medical University), Shanghai, China

**Keywords:** hepatocellular carcinoma, lenvatinib, propensity score matching (PSM), nomogram, microvascular invasion

## Abstract

**Background and Aim:**

Microvascular invasion (MVI) has been established as one of the most important contributors to the prognosis of primary hepatocellular carcinoma (HCC). The objective of this study was to investigate the potential effect of postoperative adjuvant therapy with lenvatinib on the long-term prognosis after radical resection in hepatitis B virus (HBV)-related HCC patients with MVI, as well as to predict the long-term survival based on nomograms.

**Methods:**

Data from 293 HBV-related hepatocellular carcinoma patients with histologically confirmed MVI who underwent R0 resection at Eastern Hepatobiliary Surgery Hospital (EHBH) was retrospectively analyzed. 57 patients received postoperative adjuvant therapy with lenvatinib, while 236 patients did not. The survival outcome of patients who received postoperative adjuvant lenvatinib versus those who did not was analyzed.

**Results:**

The 1-year, 2-year recurrence rates and survival rates of the lenvatinib group were improved compared to the non-lenvatinib group (15.9%, 43.2% vs 40.1%, 57.2%, P=0.002; 85.8%, 71.2% vs 69.6%, 53.3%, P=0.009, respectively). Similar findings were also observed after Propensity Score Matching (PSM) compared to non-PSM analyses The 1-year, 2-year recurrence rates and survival rates were more favorable for the lenvatinib group compared to the non-lenvatinib group (15.9%, 43.2% vs 42.1%, 57.4%, P=0.028; 85.8%, 71.2% vs 70.0%, 53.4%, P=0.024, respectively). As shown by univariate and multivariate analyses, absence of adjuvant lenvatinib treatment was identified as an independent risk factor for recurrence and survival. The established nomograms displayed good performance for the prediction of recurrence and survival, with a C-index of 0.658 and 0.682 respectively.

**Conclusions:**

Postoperative adjuvant therapy with lenvatinib was associated with improved long-term prognosis after R0 Resection in HBV-related HCC patients with MVI, which could be accurately predicted from nomograms.

## Introduction

Primary hepatocellular carcinoma (HCC) is the sixth most commonly occurring malignancy and the third leading cause of cancer-related mortality worldwide (1). Radical treatment for early and intermediate stages of HCC primarily includes hepatectomy and liver transplantation. As a result of the limited availability and exorbitant cost of liver transplantation, hepatectomy is considered as the first choice for the radical cure of HCC (2). However, the 5-year postoperative recurrence rate of HCC remains as high as 70%-80% (3, 4) The presence of microvascular invasion (MVI) indicates a more aggressive HCC, and patients in this setting may display earlier recurrence and distant metastasis. Therefore, MVI is currently considered as one of the most critical predictors of HCC recurrence (5, 6). Previous studies have shown a prevalence of MVI ranging from 15.0% to 57.1% in samples obtained from hepatectomy or liver transplantation (5).

As a novel molecular targeted agent, lenvatinib is an oral multi-kinase inhibitor that is predominantly active against VEGFR 1-3, FGFR 1-4, PDGF receptor-alpha, RET and KIT (7). In the REFLECT study, non-inferiority in overall survival rate and significant improvement in progression-free survival, time to progression, time to progression, objective response rate, and safety were demonstrated for lenvatinib compared to sorafenib in patients with advanced unresectable HCC (7). As shown by the subgroup analysis, the overall survival was substantially longer in patients with HBV-related HCC who received lenvatinib compared to those who were given sorafenib. Currently, lenvatinib is recommended as a first-line treatment for unresectable HCC in NCCN, ECMO, AASLD, EASL and Chinese clinical guidelines for the management of HCC (8–12).

Postoperative adjuvant treatments, including TACE, sorafenib and Huaier Granule, improved the long-term prognosis after radical hepatectomy in HCC patients with MVI (13–15). However, whether the postoperative adjuvant treatment with lenvatinib as anti-recurrence therapy improves the prognosis of Hepatitis B Virus–related HCC with MVI after Radical Resection has not been described.

Therefore, 57 patients who received postoperative adjuvant therapy with lenvatinib and 236 patients who did not were included in this study, with the purpose of analyzing the long-term prognosis of these two groups and establishing nomograms to predict the long-term survival of the patients.

### Patient Selection

The study enrolled 293 HBV-related HCC patients with MVI who underwent radical hepatectomy at Eastern Hepatobiliary Surgery Hospital (The Third Affiliated Hospital of People’s Liberation Army Naval Medical University) from June 1, 2019 to June 1, 2021, including 57 patients who received postoperative adjuvant therapy with lenvatinib and 236 patients who did not. This study was approved by the Institutional Ethics Committee of the hospital and each patient signed the informed consent to collect his/her data for the purpose of the study. The inclusion criteria included: 1. Pathological diagnosis of HCC; 2. Child - Pugh A or B7 (16); 3. Had not received any anti-tumor treatment before surgery; 4. R0 resection with pathological diagnosis of M1 or M2; 5. Aged 18-70 years; and 6. ECOG score of 0 or 1. The exclusion criteria included: 1. R0 resection with pathological diagnosis of M0;2. Child-Pugh beyond B7, presence of CSPH or refractory ascites; 3.Had received preoperative anti-tumor treatment; 4. Medical histories of other tumors; 5. AFP can’t decreased to the normal level as re-determined in one month after surgery; 6.Elective surgery due to tumor rupture; and 7. incomplete clinical data.

Retrospective variables included age, sex, hepatitis B virus-deoxyribonucleic acid(HBV-DNA), total bilirubin(TBIL), albumin(ALB), alanine aminotransferase(ALT), platelet count(PLT), prothrombin time(PT), neutrophil‐to‐lymphocyte ratio(NLR), alpha fetoprotein (AFP), blood transfusion, and resection margin. Tumor pathological data included maximum tumor diameter, tumor number, MVI, tumor capsule, tumor differentiation and liver cirrhosis classification. MVI was defined as the presence of cancer cell nests in portal and hepatic veins lined with endothelial cells, as well as in tumor capsular vessels (17); M1 (1–5 sites of MVI occurring in the tumor-adjacent liver tissue ≤ 1 cm away from the main tumor), M2 (> 5 MVI sites, or any MVI existing in the distant liver tissue > 1 cm away from the main tumor) (17). A wide or narrow resection margin was defined as the shortest distance ≥1 cm or <1 cm from the tumor edge to the LR plane, which was consistent to the definition described elsewhere (18–20). Early recurrence was defined as recurrence within 1 year after surgery (21, 22).

### Usage of Lenvatinib

Patients in the lenvatinib group were given oral lenvatinib (Eisai, Japan) 12 mg/d (B.W. ≥ 60 kg) or 8 mg/day (B.W. < 60 kg) on a 28-day cycle, until HCC recurrence, serious adverse events (SAE) or spontaneous withdrawal. Interruption or dose reduction was allowed to alleviate toxicities related to lenvatinib (with the dose reduced to 8 mg and 4 mg per day or 4 mg every other day). Adverse events were classified according to the National Cancer Institute Common Terminology Criteria for Adverse Events (NCI-CTCAE) v 4.0.

### Postoperative Follow-Up

All the patients received prophylactic TACE for about a month after surgery (23). Testing of AFP as a tumor marker in peripheral blood, ultrasonography, and contrast-enhanced CT or magnetic resonance imaging (MRI) of the abdomen were completed in follow-up visits which were performed every 2 months during the first 6 month and every half year thereafter. Study endpoints included overall survival (OS) and time to recurrence (TTR). OS was determined based on the duration from the date of liver resection to the date of death or the last follow-up. In contrast, TTR was calculated from the date of liver resection to that of the first HCC recurrence or the last follow-up.

### Statistical Analysis

Statistical analysis was conducted using R software version 4.0.0, (http://www.R-project.org). Continuous variables of normal distribution were expressed as mean ± standard deviation. Categorical variables were denoted with number (n) or proportion (%). Continuous variables were compared using independent samples t-test if applicable; otherwise, Mann–Whitney U test was employed. Categorical variables were compared using the Chi-square test or Fisher’s exact test if appropriate. A 1:1 propensity score matching (PSM) was performed to adjust for confounding factors between two groups. The binary logistic regression with selected variables was used to produce continuous propensity scores from 0 to 1. The nearest-neighbor match between with and without adjuvant lenvatinib patients was performed to select patients for subsequent analyses and the pairs on the propensity-score logit were then matched to within a range of 0.2 of standard deviation. OS and TTR were calculated by the Kaplan–Meier method generated by the log-rank test. Independent risk factors for OS and TTR were identified based on univariate and multivariate Cox regression analyses. As for variables with P<0.05 in univariate analysis, analyses were implemented using a multivariate Cox regression model with a positive stepwise variable selection method. The statistical significance level was set at P<0.05 for all analyses.

## Results

### Demographic Characteristics and Clinical Data

Among 896 HCC patients who underwent radical hepatic resection in our hospital, 603 patients were excluded. Two hundred and ninety-three (293) patients were enrolled (**Figure S1**), including 57 patients who received postoperative adjuvant treatment with lenvatinib and 236 patients who did not. Comparison of the clinical data of the two groups is shown in **Table 1**. Statistical differences were observed in HBV-DNA, PT, AFP and MVI. In order to eliminate potential bias induced by differences in baselines characteristics, PSM was implemented for the two groups. The lenvatinib and non-lenvatinib groups both included 57 patients after PSM (**Table 1**).

**Table 1:** Basal clinicopathological characteristics of 293 HCC patients with Microvascular Invasion with and without adjuvant Lenvatinib.

Variable	Before PSM			After PSM		
	No Lenvatinib (n = 236)	Lenvatinib (n = 57)	*P*	No Lenvatinib (n = 57)	Lenvatinib (n = 57)	*P*
**Age**	52 (21-69)	53 (20-70)	0.265	52 (21-69)	53 (20-70)	0.135
**Gender**			0.073			0.178
Female	213 (90.3)	46 (80.7)		52 (91.2)	46 (80.7)	
Male	23 (9.75)	11 (19.3)		5 (8.77)	11 (19.3)	
**HBV-DNA**, IU/mL			**0.004**			1.000
≤2000	105 (44.5)	38 (66.7)		37 (64.9)	38 (66.7)	
>2000	131 (55.5)	19 (33.3)		20 (35.1)	19 (33.3)	
**TBIL**, μmol/L			0.399			1.000
≤17	162 (68.6)	43 (75.4)		44 (77.2)	43 (75.4)	
>17	74 (31.4)	14 (24.6)		13 (22.8)	14 (24.6)	
**ALB**, g/L			0.258			0.679
≤35	8 (3.39)	4 (7.02)		2 (3.51)	4 (7.02)	
>35	228 (96.6)	53 (93.0)		55 (96.5)	53 (93.0)	
**ALT**, U/L			0.195			0.702
≤44	124 (52.5)	36 (63.2)		33 (57.9)	36 (63.2)	
>44	112 (47.5)	21 (36.8)		24 (42.1)	21 (36.8)	
**PLT**, *10^9^/ml			1.000			0.178
≤100	44 (18.6)	11 (19.3)		5 (8.77)	11 (19.3)	
>100	192 (81.4)	46 (80.7)		52 (91.2)	46 (80.7)	
**PT**, S			**0.032**			1.000
≤13	183 (77.5)	52 (91.2)		53 (93.0)	52 (91.2)	
>13	53 (22.5)	5 (8.77)		4 (7.02)	5 (8.77)	
**NLR**			0.614			0.064
≤2.4	159 (67.4)	41 (71.9)		29 (50.9)	37 (64.9)	
>2.4	77 (32.6)	16 (28.1)		28 (49.1)	20 (35.1)	
**AFP**, ng/mL			**0.007**			1.000
≤400	88 (37.3)	33 (57.9)		33 (57.9)	33 (57.9)	
>400	148 (62.7)	24 (42.1)		24 (42.1)	24 (42.1)	
**Transfusion**			0.378			0.164
No	157 (66.5)	42 (73.7)		34 (59.6)	42 (73.7)	
Yes	79 (33.5)	15 (26.3)		23 (40.4)	15 (26.3)	
**Tumor diameter**, cm			0.515			1.000
≤5	54 (22.9)	16 (28.1)		17 (29.8)	16 (28.1)	
>5	182 (77.1)	41 (71.9)		40 (70.2)	41 (71.9)	
**Tumor number**			0.389			0.823
1	200 (84.7)	45 (78.9)		43 (75.4)	45 (78.9)	
≥2	36 (15.3)	12 (21.1)		14 (24.6)	12 (21.1)	
**Microvascular invasion**			**0.049**			1.000
M1	168 (71.1)	31 (54.3)		33 (57.8)	31 (54.3)	
M2	68 (28.9)	26 (45.7)		24 (42.2)	26 (45.7)	
**Tumor capsule**			0.770			1.000
Complete	129 (54.7)	33 (57.9)		32 (56.1)	33 (57.9)	
Incomplete	107 (45.3)	24 (42.1)		25 (43.9)	24 (42.1)	
**Margin**			0.283			0.430
Narrow	91 (38.6)	17 (29.8)		22 (38.6)	17 (29.8)	
Wide	145 (61.4)	40 (70.2)		35 (61.4)	40 (70.2)	
**Edmondson-Steiner grade**			0.096			1.000
I-II	10 (4.24)	6 (10.5)		5 (8.77)	6 (10.5)	
III-VI	226 (95.8)	51 (89.5)		52 (91.2)	51 (89.5)	
**Cirrhosis**			1.000			0.254
No	85 (36.0)	20 (35.1)		27 (47.4)	20 (35.1)	
Yes	151 (64.0)	37 (64.9)		30 (52.6)	37 (64.9)	

Bold values indicate statistical significance (P < 0.05).

HCC, Hepatocellular Carcinoma; PSM, propensity score matching. HBV-DNA, hepatitis B virus-deoxyribonucleic acid; TBIL, total bilirubin; ALB, albumin; ALT, Alanine aminotransferase; PT, Prothrombin time; PLT, platelet; NLR, neutrophil‐to‐lymphocyte ratio; AFP, alpha fetoprotein.

### Adverse Events of Lenvatinib

In the lenvatinib group, all the patients tolerated the oral treatment with lenvatinib for at least three cycles, although 18 patients had their dose reduced due to adverse reactions of CTCAE grade 2, and 5 patients discontinued lenvatinib treatment after the dose reduction. The occurrence of adverse reactions in the lenvatinib group is presented in **Table 2**. The overall incidence of adverse reactions was 87.7% (50/57). Hypertension was identified to be the most common adverse reaction, and no fatal adverse event was reported. The most severe adverse events were 5 events of CTCAE grade 3.

**Table 2:** Adverse events in treatment of adjuvant Lenvatinib after radical resection and their corresponding common terminology criteria for adverse events (CTCAE) grade.

Adverse events	Adjuvant Lenvatinib (n = 57)			
	All	Grade 1	Grade 2	Grade 3
Hypertension	20	10	7	3
PPES	13	7	5	1
Diarrhoea	19	9	8	2
Fatigue	15	9	6	0
Decreased appetite	16	7	9	0
Hypothyroidism	6	4	2	0
DILI	17	10	6	1
Others	10	7	3	0

PPES, palmar-plantar erythrodysesthesia syndrome; DILI, drug-induced liver injury.

### Survival Analysis

**Figure 1:**
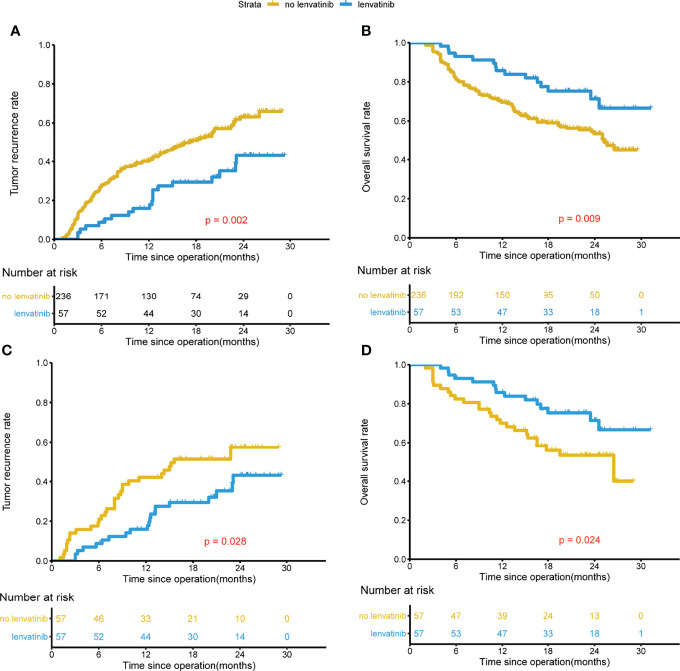
Kaplan-Meier analysis for predicting survival in HCC patients with MVI after radical resection. Before PSM, TTR and OS for patients with and without adjuvant Lenvatinib **(A, B)**. After PSM, TTR and OS for patients with and without adjuvant Lenvatinib **(C, D)**.

**Figure 2:**
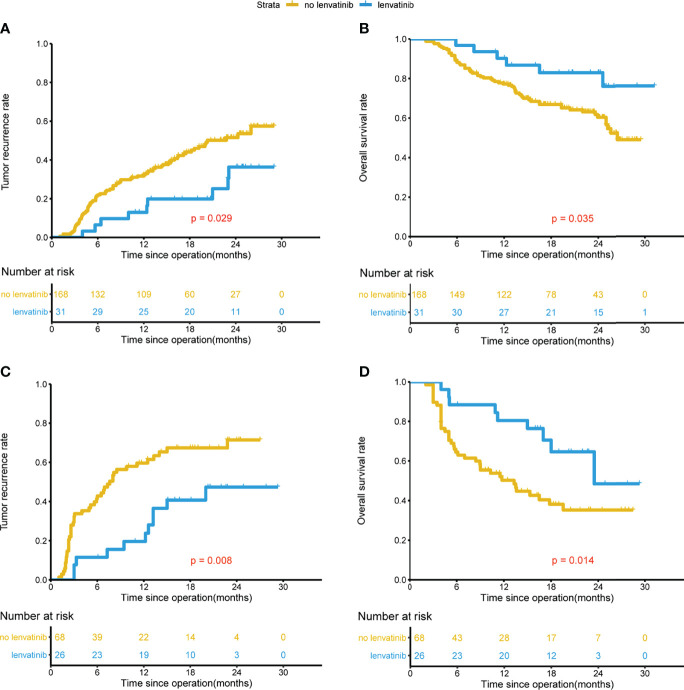
Kaplan-Meier analysis for predicting survival in HCC patients with MVI beings M1 and M2 after radical resection. MVI beings M1, TTR and OS for patients with and without adjuvant Lenvatinib **(A, B)**. MVI beings M2, TTR and OS for patients with and without adjuvant Lenvatinib **(C, D)**.

The median follow-up was 22.6 months for the lenvatinib group, and 22.4 months for the non-lenvatinib group. Before PSM, both the TTR and OS in the lenvatinib group were significantly improved compared to those in the non-lenvatinib group (1-year and 2-year recurrence rates were 15.9%, 43.2% and 40.1%, 57.2% respectively, P=0.002; 1-year and 2-year survival rates were 85.8%, 71.2% and 69.6%, 53.6% respectively, P=0.009) (**Figures 1A, B**). After PSM, similar results were found compared to those before PSM (1-year and 2-year recurrence rates were 15.9%, 43.2% and 42.1%, 57.6% respectively, P=0.028; 1-year and 2-year survival rates were 85.8%, 71.2% and 70.0%, 53.4% respectively, P=0.024) (**Figures 1C, D**). In the group with MVI beings M1, lenvatinib group had better TTR and OS than non-lenvatinib group (**Figures 2A, B**), Similar results were noted in the group with MVI being M2 (**Figures 2C, D**).

Among the 57 patients in the lenvatinib group, 20 (35.1%) patients relapsed, including 9 patients with early recurrence and 11 patients with late recurrence. In the non-lenvatinib group, 124/236 (52.5%) patients relapsed, including 94 patients with early recurrence and 30 patients with late recurrence. Statistical differences were observed between the two groups in the number of patients with recurrence and the proportion of early recurrences (P=0.026, P=0.010). Among the 124 patients in the non-lenvatinib group, 104 patients had intrahepatic recurrence, 8 patients had extrahepatic recurrence, and 12 patients had both intrahepatic and extrahepatic recurrence. Among the 20 patients with recurrence in the lenvatinib group, 16 patients had intrahepatic recurrence, 2 patients had extrahepatic recurrence, and 2 patient had both intrahepatic and extrahepatic recurrence. There was no statistical difference in the recurrence pattern between the two groups (P=0.785) (**Table 3**). After PSM, the incidence of early recurrence in the lenvatinib group were significantly lower compared to those in the non-lenvatinib group(P=0.038).

**Table 3:** Patterns of recurrence in HCC with Microvascular Invasion with and without adjuvant Lenvatinib.

Parameters	Before PSM (n, %)			After PSM (n, %)		
	No Lenvatinib (n = 236)	Lenvatinib (n = 57)	*P*	No Lenvatinib (n = 57)	Lenvatinib (n = 57)	*P*
**No. of recurrent cases**	124 (52.5)	20 (35.1)	**0.026**	31 (54.4)	20 (35.1)	0.059
**Time to recurrence**, months*			**0.010**			**0.038**
≤12	94 (75.8)	9 (45.0)		24 (77.4)	9 (47.3)	
>12	30 (24.2)	11 (55.0)		7 (22.6)	11 (52.7)	
Type of recurrence**			0.785			1.000
Intrahepatic	104 (84.0)	16 (80)		24 (77.4)	16 (80)	
Extrahepatic	8 (6.4)	2 (10)		4 (12.9)	2 (10)	
Intra- plus extrahepatic	12 (9.6)	2 (10)		3 (10.7)	2 (10)	

Bold values indicate statistical significance (P < 0.05). HCC, Hepatocellular Carcinoma; PSM, propensity score matching.

### Risk Factors for Poor TTR and OS

Before PSM, univariate analysis and multivariate analysis showed AFP>400ng/ml (P=0.025), multiple tumors(P=0.006), MVI being M2 (P<0.001), narrow resection margin (P<0.001) and absence of adjuvant lenvatinib (P=0.001) were identified as independent risk factors for postoperative recurrence. HBV-DNA>2000 IU/mL (P=0.023), AFP>400 ng/mL (P=0.028), multiple tumors (P=0.002), MVI being M2 (P<0.001), narrow resection margin (P<0.001) and postoperative adjuvant lenvatinib (P=0.002) were identified as independent risk factors for postoperative survival (**Tables 4**, **5**). After PSM, NLR>2.4 (P=0.022), MVI being M2 (P=0.019), narrow resection margin (P=0.021)and absence of adjuvant lenvatinib (0.039) were identified as independent risk factors for postoperative recurrence; and NLR>2.4 (P=0.010), MVI being M2 (P=0.017), narrow resection margin (P=0.024)and absence of adjuvant lenvatinib (0.048) were found to be independent risk factors for postoperative long-term survival (**Tables 6**, **7**).

**Table 4:** Univariate Cox-regression analysis for predicting TTR and OS in 293 HCC patients with Microvascular Invasion with and without adjuvant Lenvatinib before PSM.

Variable	Univariate Analysis (TTR)			Univariate Analysis (OS)		
	HR	95%CI	*P*	HR	95%CI	*P*
**Age**, years	0.99	0.97-1.00	0.117	0.99	0.97-1.00	0.108
**Gender**, Male vs. Female	0.85	0.50-1.45	0.545	0.79	0.43-1.43	0.435
**HBV-DNA**, IU/mL >2000 vs. ≤2000	1.68	1.21-2.35	**0.002**	1.89	1.30-2.73	**0.001**
**TBIL**, µmol/L >2000 vs. ≤2000	0.97	0.68-1.39	0.869	0.88	0.59-1.31	0.518
**ALB, g/L** >35 vs. ≤35	1.75	0.65-4.74	0.268	1.38	0.51-3.74	0.528
**ALT,** U/L >44 vs. ≤44	0.89	0.64-1.23	0.472	0.80	0.56-1.16	0.237
**PLT,** ×10^9^/L >100 vs. ≤100	0.92	0.61-1.39	0.687	0.92	0.59-1.43	0.703
**PT**, seconds >13 vs. ≤13	0.91	0.59-1.38	0.646	1.08	0.69-1.69	0.733
**NLR** >2.4 vs. ≤2.4	1.66	1.18-2.32	**0.003**	1.69	1.17-2.45	**0.005**
**AFP**, ng/mL >400 vs. ≤400	1.63	1.16-2.29	**0.005**	1.74	1.19-2.55	**0.005**
**Transfusion** Yes vs. no	1.16	0.82-1.63	0.399	1.03	0.71-1.51	0.870
**Tumor diameter**, cm >5 vs. ≤5	1.52	1.00-2.31	0.051	1.54	0.97-2.46	0.067
**Tumor number** Multiple vs. Single	1.63	1.07-2.46	**0.021**	1.90	1.23-2.94	**0.004**
**Microvascular invasion** M2 vs. M1	1.83	1.31-2.56	**<0.001**	2.00	1.38-2.88	**<0.001**
**Tumor capsule** Incomplete vs. Complete	0.79	0.56-1.10	0.161	0.79	0.55-1.14	0.203
**Margin** Wide vs. Narrow	0.54	0.39-0.76	**<0.001**	0.52	0.36-0.75	**<0.001**
**Edmondson-Steiner grade** III-VI vs. I-II	0.93	0.49-1.77	0.826	1.68	0.68-4.11	0.258
**Cirrhosis** Yes vs. No	1.31	0.93-1.86	0.127	1.28	0.87-1.88	0.205
**Lenvatinib** Yes vs. No	0.52	0.32-0.83	**0.006**	0.49	0.29-0.85	**0.011**

Bold values indicate statistical significance (P < 0.05). HCC, Hepatocellular Carcinoma; PSM, propensity score matching; OS, overall survival; TTR, time to recurrence; HBV-DNA, hepatitis B virus-deoxyribonucleic acid; TBIL, total bilirubin; ALB, albumin; ALT, Alanine aminotransferase; PT, Prothrombin time; PLT, platelet; NLR, neutrophil‐to‐lymphocyte ratio; AFP, alpha fetoprotein.

**Table 5:** Multivariate Cox-regression analysis for predicting TTR and OS in 293 HCC patients with Microvascular Invasion with and without adjuvant Lenvatinib before PSM.

Variable	Multivariable Analysis (TTR)			Multivariable Analysis (OS)		
	HR	95%CI	P	HR	95%CI	P
**HBV-DNA**, IU/mL >2000 vs. ≤2000	–	–	–	1.56	1.06-2.29	**0.023**
**AFP**, ng/mL >400 vs. ≤400	1.50	1.05-2.15	**0.025**	1.56	1.05-2.33	**0.028**
**Tumor number** Multiple vs. Single	1.83	1.19-2.81	**0.006**	2.07	1.31-3.25	**0.002**
**Microvascular invasion** M2 vs. M1	2.22	1.54-3.22	**<0.001**	2.37	1.59-3.55	**<0.001**
**Margin** Wide vs. Narrow	0.50	0.35-0.71	**<0.001**	0.47	0.32-0.69	**<0.001**
**Lenvatinib** Yes vs. No	0.44	0.27-0.72	**0.001**	0.42	0.24-0.73	**0.002**

Bold values indicate statistical significance (P < 0.05). OS, overall survival; TTR, time to recurrence; HCC, Hepatocellular Carcinoma; PSM, propensity score matching. HBV-DNA, hepatitis B virus-deoxyribonucleic acid; AFP, alpha fetoprotein.

**Table 6:** Univariate Cox-regression analysis for predicting TTR and OS in 114 HCC patients with Microvascular Invasion with and without adjuvant Lenvatinib after PSM.

Variable	Univariate Analysis (TTR)			Univariate Analysis (OS)		
	HR	95%CI	*P*	HR	95%CI	*P*
**Age**, years	0.99	0.97-1.01	0.312	0.99	0.96-1.01	0.291
**Gender**, Male vs. Female	0.64	0.26-1.62	0.350	0.65	0.23-1.83	0.415
**HBV-DNA**, IU/mL Negative vs. Positive	1.46	0.83-2.57	0.187	1.42	0.76-2.68	0.273
**TBIL**, µmol/L >2000 vs. ≤2000	1.32	0.70-2.48	0.388	1.21	0.59-2.47	0.605
**ALB, g/L** >35 vs. ≤35	0.89	0.28-2.87	0.848	0.71	0.22-2.31	0.573
**ALT,** U/L >44 vs. ≤44	0.77	0.43-1.37	0.380	0.64	0.33-1.24	0.187
**PLT,** ×10^9^/L >100 vs. ≤100	0.85	0.40-1.81	0.674	0.71	0.33-1.55	0.396
**PT**, seconds >13 vs. ≤13	1.81	0.77-4.26	0.171	2.09	0.88-4.97	0.096
**NLR** >2.4 vs. ≤2.4	2.62	1.50-4.59	**0.001**	2.78	1.48-5.24	**0.001**
**AFP**, ng/mL >400 vs. ≤400	2.10	1.21-3.65	**0.009**	1.73	0.93-3.22	0.081
**Transfusion** Yes vs. no	1.64	0.94-2.86	0.079	1.45	0.78-2.70	0.242
**Tumor diameter**, cm >5 vs. ≤5	1.04	0.56-1.93	0.904	0.87	0.45-1.69	0.679
**Tumor number** Multiple vs. Single	1.41	0.75-2.65	0.288	1.54	0.77-3.08	0.222
**Microvascular invasion** M2 vs. M1	2.06	1.18-3.59	**0.011**	2.07	1.11-3.86	**0.022**
**Tumor capsule** Incomplete vs. Complete	0.60	0.33-1.07	0.084	0.69	0.36-1.30	0.246
**Margin** Wide vs. Narrow	0.55	0.31-0.96	**0.035**	0.51	0.27-0.94	**0.031**
**Edmondson-Steiner grade** III-VI vs. I-II	0.61	0.28-1.29	0.193	0.91	0.35-2.34	0.846
**Cirrhosis** Yes vs. No	1.60	0.89-2.87	0.114	1.43	0.75-2.74	0.273
**Lenvatinib** Yes vs. No	0.54	0.31-0.94	**0.030**	0.49	0.26-0.92	**0.026**

Bold values indicate statistical significance (P < 0.05). HCC, Hepatocellular Carcinoma; PSM, propensity score matching; OS, overall survival; TTR, time to recurrence; HBV-DNA, hepatitis B virus-deoxyribonucleic acid; TBIL, total bilirubin; ALB, albumin; ALT, Alanine aminotransferase; PT, Prothrombin time; PLT, platelet; NLR, neutrophil‐to‐lymphocyte ratio; AFP, alpha fetoprotein.

**Table 7:** Multivariate Cox-regression analysis for predicting TTR and OS in 114 HCC patients with Microvascular Invasion with and without adjuvant Lenvatinib after PSM.

Variable	Multivariable Analysis (TTR)			Multivariable Analysis (OS)		
	HR	95%CI	P	HR	95%CI	P
**NLR** >2.4 vs. ≤2.4	1.98	1.10-3.57	**0.022**	2.34	1.23-4.46	**0.010**
**Microvascular invasion** M2 vs. M1	2.00	1.12-3.56	**0.019**	2.18	1.15-4.13	**0.017**
**Margin** Wide vs. Narrow	0.51	0.28-0.90	**0.021**	0.49	0.26-0.91	**0.024**
**Lenvatinib** Yes vs. No	0.55	0.31-0.97	**0.039**	0.52	0.28-0.99	**0.048**

Bold values indicate statistical significance (P < 0.05). OS, overall survival; TTR, time to recurrence; HCC, Hepatocellular Carcinoma; PSM, propensity score matching. NLR, neutrophil‐to‐lymphocyte ratio.

### Prognostic Nomograms for TTR and OS Before PSM

**Figure 3:**
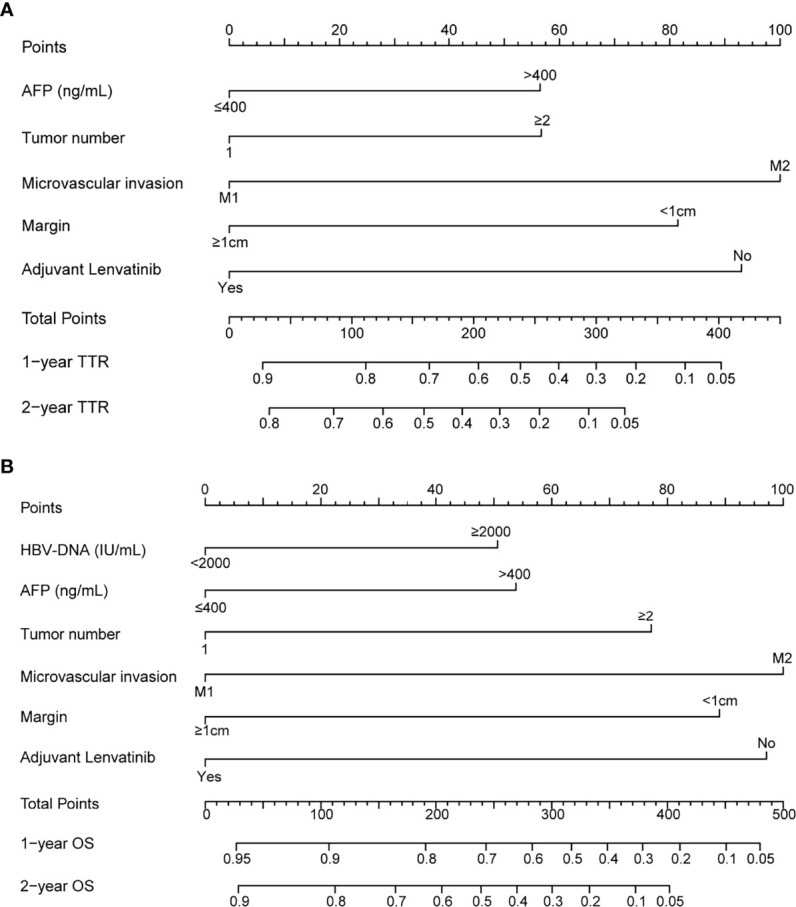
Nomogram for survival of HCC patients with MVI after radical resection. adjuvant Lenvatinib-related nomograms for TTR **(A)** and OS **(B)**.

**Figure 4:**
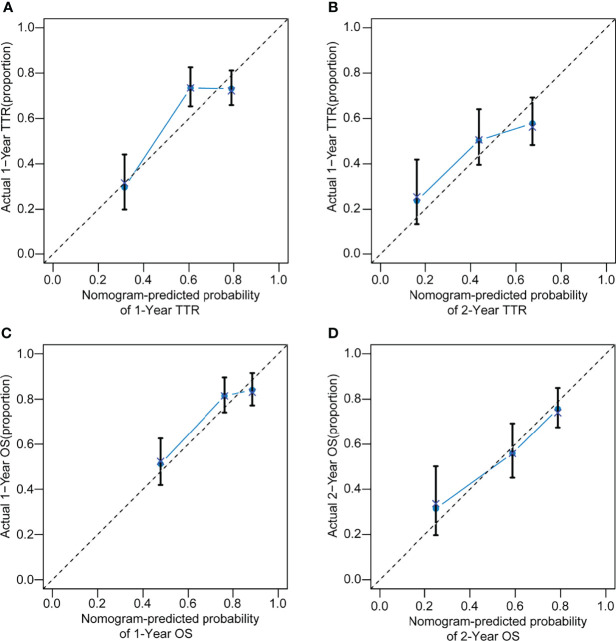
The calibration curve for predicting TTR at 1 years **(A)**, 2 years **(B)** and OS at 1 years **(C)**, 2 years **(D)** in HCC patients with MVI after radical resection.

Based on the independent risk factors associated with recurrence and survival identified before PSM, nomograms were established (**Figures 3A, B**). The C-index were 0.658 and 0.682 for TTR and OS prediction. As shown in the calibration curves for 1-year, 2-year recurrence and survival rates, nomogram predictions and actual observations appeared to be highly comparable (**Figure 4**).

## Discussion

HCC is one of the most commonly diagnosed malignancies worldwide (24). Over the past several decades, treatment of HCC has evolved to a great extent. Surgical resection has been recognized as the first-line treatment for HCC in its early and intermediate stages. Unfortunately, the recurrence rate remains high after resection and the long-term survival is found to be very low, especially in patients with vascular invasion (25, 26). How to delay the recurrence of HCC patients remains to be a challenge in the treatment of HCC.

This study aimed to investigate the safety and prognosis of postoperative adjuvant lenvatinib anti-recurrence therapy in HBV-related HCC patients with MVI to guide rational clinical decision making. In this study, adjuvant lenvatinib after radical hepatectomy reduced the early recurrence rate and prolonged the OS. Similar results were obtained after bias due to baseline differences was eliminated by PSM. Adverse events of adjuvant lenvatinib treatment were generally manageable. The present study is the first to describe the use of lenvatinib as an adjuvant therapy to reduce the risk of postoperative recurrence and improve long-term survival outcomes in HBV-related HCC patients with MVI.

MVI is acknowledged as an expression of aggressive biological behavior of the tumor and is currently one of the most critical factors predicting HCC recurrence (5, 27, 28).The presence of MVI is a key determinant of recurrence and prognosis after hepatectomy for early-stage HCC. Improving the prognosis of MVI-positive HCC represents a major challenge for liver oncology surgery. As there is no effective way to diagnose MVI before surgery, adjuvant therapy, such as postoperative adjuvant TACE, and radiotherapy, has been used after hepatectomy to improve the prognosis of this group of patients (14, 23, 29, 30). Before the introduction of lenvatinib, sorafenib was the first and only molecule-targeted drug approved for HCC treatment, and the effects of sorafenib on the prevention of HCC recurrence after liver resection have been evaluated (13, 31). Huang et al. demonstrated both improved tumor-free survival and OS with postoperative adjuvant sorafenib in MVI-positive patients (31). In another study described by Zhang et al., 147 HCC patients with MVI who received adjuvant sorafenib after R0 resection showed 1-, 3-, and 5-year tumor-free survival and OS rates of 66.0%, 40.0%, 24.0% and 70.0%, 54.0%, 43.0%, respectively, which were significantly improved compared with those observed in patients who had not received postoperative adjuvant sorafenib (P=0.029, P=0.003, respectively). Similar results were described after PSM (13).

Lenvatinib has been found to have non-inferior efficacy to sorafenib in untreated advanced HCC (7). It is considered the best treatment option for HBV-related HCC due to the lowest HR compared with sorafenib (HR 0.83, 95% CI 0.68-1.01), although the reasons for the divergent responses by viral etiology remain unclear (32). Several studies have reported that lenvatinib is more effective than sorafenib in treating HCC with macrovascular invasion, this may be owing to the potent activity against FGFR1–4 is a distinctive feature of lenvatinib, compared with sorafenib (33–36). According to the HCC guidelines, lenvatinib is recommended only for HCC with macrovascular invasion (10). Whether to administer lenvatinib for HCC with MVI remains controversial, even though MVI is a key factor for recurrence and metastasis after surgery. We, therefore, designed this study to investigate whether postoperative adjuvant lenvatinib treatment improves prognosis in HBV-related HCC patients with MVI.

Adjuvant treatment with lenvatinib has been shown to inhibit tumor recurrence and metastasis after liver transplantation in HBV-related HCC patients with a high risk of recurrence (37). In a study described by Han, 14 HCC patients with a high risk of recurrence who received liver transplantation followed by adjuvant lenvatinib and 9 such patients who did not receive adjuvant lenvatinib treatment were included. The results showed that the progression-free survival of the lenvatinib group was significantly better than the control group (P=0.04) (37). There has been no report elaborating the effect of adjuvant lenvatinib on the long-term survival of HCC in patients who underwent liver resection. In this study, 57 patients receiving adjuvant lenvatinib were included for recurrence and survival analysis. The results indicated that the 1-year, 2-year recurrence rates and survival rates were more favorable for the lenvatinib group compared to the non-lenvatinib group (15.9%, 43.2% vs 40.1%, 57.2%, P=0.002; 85.8%, 71.2% vs 69.2%, 53.3%, P=0.009, respectively). After elimination of potential bias induced by differences in baselines characteristics, PSM was implemented and similar findings were observed compared to those before PSM. The 1-, 2-year recurrence rates and survival rates of the lenvatinib group were improved compared to the non-lenvatinib group (15.9%, 43.2% vs 42.1%, 57.4%, P=0.028; 85.8%, 71.2% vs 70.0%, 53.4%, P=0.024, respectively). As shown by univariate and multivariate analyses, absence of adjuvant lenvatinib treatment has been identified as independent risk factors for recurrence and survival. Additionally, nomograms were established based on these independent risk factors, which displayed good prediction performance.

This study has some limitations. First of all, this study is a single-center retrospective trial, and multi-center, large sample studies are still needed to further confirm the findings. Next, the data on 3-year survival was not available due to relatively short duration of follow-up. We will increase our sample size and extend the duration of follow-up in our future studies. Additionally, this study was conducted in China and included only patients with underlying condition of HBV infection. Thus the findings warrant further validation from study cohorts with hepatitis C virus infection or alcoholic or non-alcoholic fatty liver disease as the dominant pathology of HCC.

## Conclusions

In conclusion, the study demonstrated that postoperative adjuvant lenvatinib therapy could improve the long-term prognosis after R0 resection in HBV-related HCC patients with MVI, which could be accurately predicted based on the established nomograms. However, the findings of this study warrant further validation by conducting multicenter randomized controlled trials of large sample size.

## Data Availability Statement

The raw data supporting the conclusions of this article will be made available by the authors, without undue reservation.

## Ethics Statement

This study was approved by the Institutional Ethics Committee of the hospital and each patient signed the informed consent to collect his/her data for the purpose of the study.

## Author Contributions

Study concept and design: SB, JL, FX and YS. Acquisition, analysis, or interpretation of data: SB, JL and MS. Statistical analysis: SB. Critical revision of the manuscript for important intellectual content: FX and YS. All authors contributed to the article and approved the submitted version.

## Conflict of Interest

The authors declare that the research was conducted in the absence of any commercial or financial relationships that could be construed as a potential conflict of interest.

## Publisher’s Note

All claims expressed in this article are solely those of the authors and do not necessarily represent those of their affiliated organizations, or those of the publisher, the editors and the reviewers. Any product that may be evaluated in this article, or claim that may be made by its manufacturer, is not guaranteed or endorsed by the publisher.

## Supplementary Material

Supplementary Figure 1 Flow Chart of patient’s inclusion
