# Supplementary figures and images for: Prognostic Nomograms Combined Adjuvant Lenvatinib for Hepatitis B Virus–related Hepatocellular Carcinoma With Microvascular Invasion After Radical Resection
Source: Front Oncol. 2022 Jul 11;12:919824. doi: 10.3389/fonc.2022.919824 (PMC9309730; doi:10.3389/fonc.2022.919824)

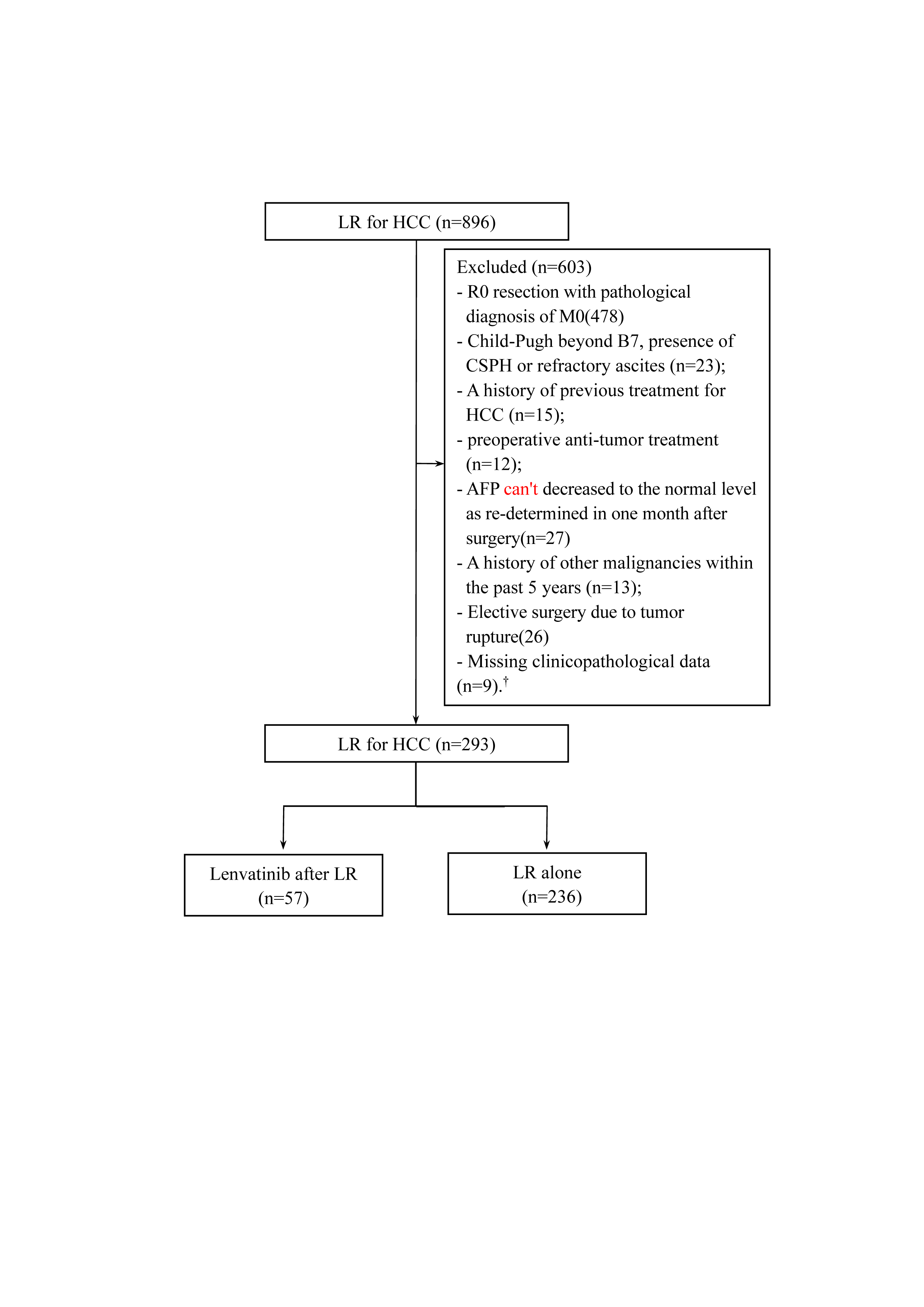

Supplement: Supplementary Figure 1 — Flow Chart of patient’s inclusion [file Image_1.tif]
